# Mitochondrial genome of *Lonicera macranthoides*: features, RNA editing, and insights into male sterility

**DOI:** 10.3389/fpls.2024.1520251

**Published:** 2025-01-10

**Authors:** Zhong Chen, Wei Zhuo, Yuqi Wang, Junpeng Qi, Li Liu, Sheng’E. Lu, Han Wang, Tao Sun, Liqiang Wang, Fengming Ren

**Affiliations:** ^1^ Bio-resource Research and Utilization Joint Key Laboratory of Sichuan and Chongqing, Chongqing Institute of Medicinal Plant Cultivation, Nanchuan, Chongqing, China; ^2^ Chongqing Customs Technology Center, Shapingba, Chongqing, China; ^3^ College of Pharmacy, Heze University, Heze, Shandong, China; ^4^ School of Chinese Materia Medica, Chongqing College of Traditional Chinese Medicine, Bishan, Chongqing, China

**Keywords:** Caprifoliaceae, *Lonicera macranthoides*, mitogenome, RNA editing, CMS

## Abstract

**Introduction:**

Mitochondria are essential organelles that provide energy for plants. They are semi-autonomous, maternally inherited, and closely linked to cytoplasmic male sterility (CMS) in plants. *Lonicera macranthoides*, a widely used medicinal plant from the Caprifoliaceae family, is rich in chlorogenic acid (CGA) and its analogues, which are known for their antiviral and anticancer properties. However, studies on the mitogenome of *L. macranthoides* still remain limited.

**Methods:**

The mitochondrial DNA contained in the whole genome DNA was extracted from a male sterile cultivar of *L. macranthoides*, named ‘Yulei 1’. Next-generation sequencing (NGS) and third-generation sequencing (TGS) technologies were combined to obtain the mitogenome. RNA editing events were identified by integrating the mitogenome data with RNA sequencing data from leaf, stem, and flower tissues. The potential causes of male sterility in ‘Yulei 1’ were analyzed based on the loss of functional genes, mitogenome rearrangements, RNA editing events, and open reading frames (ORFs).

**Results and discussion:**

The complete mitogenome of *L. macranthoides* ‘Yulei 1’ was obtained for the first time, with a length of 1,002,202 bp. It contains 48 protein-coding genes (PCGs), 26 tRNA genes, and 3 rRNA genes. Additionally, 79 simple sequence repeats (SSRs), 39 tandem repeats, and 99 dispersed repeats were identified. Among these, two direct repeats (RP1a/1b, RP2a/2b) and two inverse repeats (RP3a/b, RP4a/b) may facilitate mitogenome recombination. Gene transfer analysis revealed that 4.36% and 21.98% of mitogenomic sequences mapped to the chloroplast and nuclear genomes, respectively. Phylogenetic analysis indicated that *L. macranthoides* is closest to *L. japonica* at the mitogenome level. Notably, RNA editing events varied across different plant tissues, with 357 editing sites in 30 PCGs in leaves, 138 sites in 24 PCGs in flowers, and 68 sites in 13 PCGs in stems. Finally, all indications of CMS in the mitogenome were screened, including the detection of ORFs, and the findings showed no mutations in the mitogenome that would explain the sterility of ‘Yulei 1’. Overall, our study provides a complete mitogenome of *L. macranthoides*, which will aid in its genetic marker exploration, evolutionary relationship analysis, and breeding programs.

## Introduction

*Lonicera macranthoides* belongs to the genus *Lonicera* within the Caprifoliaceae family. It has been widely used and cultivated as a medicinal plant in Asian countries for centuries (Sun et al., 2021). Historically, the flowers of *L. macranthoides* have been employed to treat heat-related illnesses, cardiovascular diseases, and inflammation. In recent years, these flowers have also shown potential in preventing emerging infectious diseases, such as SARS coronavirus, H1N1 influenza, and COVID-19 (Wang et al., 2011; Hu et al., 2021; Lee et al., 2021). Chlorogenic acids (CGAs) are natural polyphenolic compounds and the main active ingredients in the flowers of *L. macranthoides* (Zhou and Tong, 2003). Previous studies have shown that chlorogenic acids exhibit strong biological activities, including anti-inflammatory (Hammer and Birt, 2014), antibacterial (Wang et al., 2015), antiviral (Chen et al., 2016), radical scavenging (Chen et al., 2016), hypoglycemic (Bassoli et al., 2008), hypolipidemic (Sung et al., 2015), and hypotensive effects (Onakpoya et al., 2015). These findings partially clarified the pharmacological mechanism of *L. macranthoides.* Precisely for the broad applications of CGAs, *L. macranthoides* meet an increasing demand as a mainly natural source of CGAs. The nuclear genome and chloroplast genome of *L. macranthoides* have been studied (Hu et al., 2018; Yin et al., 2023). But the mitogenome of *L. macranthoides* has not been reported yet. Perhaps for this reason, the research field of the mitochondrion of *L. macranthoides* is still nearly blank.

Mitochondria are widely believed to have originated from a bacterial endocytosis event (Gray et al., 1999; Wang et al., 2024). Subsequently, mitochondria coordinate with the nuclei to regulate energy supply in eukaryotic cells. As important cytoplasmic organelles, mitochondria have unique genomes that differ from nuclear genomes in terms of evolutionary conservation and diversity. The number of genes in mitochondria is relatively stable, but the size of the mitogenome can vary by more than 100-fold between species (Galtier, 2011; Gualberto and Newton, 2017). The published mitogenome typically exists in a circular form, similar to plasmids in prokaryotes. However, other shapes, such as rod-shaped and branched forms, have also been reported. These conformations can change through rearrangements (Maréchal and Brisson, 2010). Additionally, RNA editing events are frequently observed in various mitogenomes, playing crucial roles in gene function (Galtier, 2011). These characteristics reflect the diversity of mitogenomes in heredity and regulation.

Mitochondria are semi-autonomous and maternally inherited organelles. Their genetic materials often exchange with nuclear genomes, and/or receive input from the chloroplast genomes to drive mitogenome evolution. This is especially evident in flowering plants, where the mitogenome evolves at a faster rate (Cho et al., 2004; Noutsos et al., 2005; Zwonitzer et al., 2024). During the evolutionary process of mitogenome, some abortive individuals may arise, such as the “WA, Wild Abortive” phenotype in rice, which is precisely caused by the emergence of new expressed genes resulting from structural variations in the mitogenome (Liu et al., 2007). Numerous studies have indicated that cytoplasmic male sterility (CMS) is usually associated with abnormal mitochondrial performance (Liu et al., 2007; Cheng et al., 2021). Genome recombination, interaction between mitochondrion and nucleus, aberrant RNA editing, and the accumulation of toxic products (encoded by open reading frames, ORFs) can all contribute to plant CMS (Chase, 2007; Chen et al., 2017). To date, benefit the progress in sequencing technology and the increasing availability of mitogenomic data, the molecular mechanisms underlying CMS have been elucidated in many species. Moreover, based on these mitogenomes, many molecular markers were developed and phylogenetic relationships were clarified (Handa, 2003; Cheng et al., 2021).

In this study, we integrated next-generation sequencing (NGS) and third-generation sequencing (TGS) data to assemble the mitogenome of *L. macranthoides* cultivar ‘Yulei 1’, whose pollen exhibits germinating abnormalities and classic characteristics of male sterility. We determined the features of this mitogenome and detected sequences transferred among the mitochondrion, nucleus, and chloroplast. We also clarified the phylogenetic relationship of *L. macranthoides*. Specifically, by combining mitogenomic and RNA sequencing data, we analyzed RNA editing events in mitochondrial protein-coding genes (PCGs) across flower, stem, and leaf tissues. Finally, we aimed to identify the reasons for the sterility of ‘Yulei 1’ in its mitogenome, including the detection of ORFs. Overall, our study seeks to enrich the genetic resources of *L. macranthoides* and provide new perspectives on its phylogenetic relationships and future breeding programs at the mitogenome level.

## Materials and methods

### Plant materials, DNA extraction, and sequencing

*L. macranthoides* samples were provided by the Chongqing Institute of Medicinal Plant Cultivation (CQIMPC) and identified by researcher Zhengyu Liu. The samples included the sterile cultivar ‘Yulei 1’ and a fertile wild type, both primarily cultivated in Xiushan County, Chongqing, China (108°58′ E, 28°27′ N). Fresh leaves were collected and immediately frozen in liquid nitrogen for further experiments. Total genomic DNA was extracted using the cetyltrimethylammonium bromide (CTAB) method with minor modifications as previously reported (Chen et al., 2023). A DNA library with an insert size of 350 bp was constructed using the NEBNext^®^ library preparation kit (New England Biolabs, USA) and sequenced on the HiSeq Xten PE150 platform at BioMaker (Wuhan, China). NGS yielded 46.17 Gb raw bases, from which 46.07 Gb clean bases were retained, resulting in an effective rate of 99.78%. The Q20 score was 97.95%, the Q30 score was 93.14%, and the GC content was 37.61% (**Supplementary Table S1**). Raw data were deposited in GenBank (Accession No. SRR30207619).

The SQK-LSK109 linker kit (Oxford Nanopore Technologies, UK) was used to construct a long fragment DNA library, followed by high-throughput sequencing on the Nanopore PromethION platform (BioMaker, China). TGS produced 26.53 Gb raw bases, all of which were retained as clean bases. The mean length of the sequences was 20,054 bp, the maximum length was 62,526 bp, and the N50 length was 20,270 bp (**Supplementary Table S1**). Raw data were deposited in GenBank (Accession No. SRR30207658).

### Genome assembly and annotation

The NGS data was assembled with GetOrganelle (Jin et al., 2020), while the TGS data was assembled using Canu (Koren et al., 2017). Each assembled contig was identified and screened for mitochondrial origin using BLAST against the GenBank database (https://blast.ncbi.nlm.nih.gov/). The resulting mitochondrial contigs served as references to filter the NGS and TGS data. Then, the filtered data was assembled into the *L. macranthoides* mitogenome using Unicycler (Phillippy et al., 2017). The assembly draft (**Supplementary Figure S1**) was visualized with Bandage (Wick et al., 2015), yielding a circularized contig representing the mitogenome. The GE-Seq tool (https://chlorobox.mpimp-golm.mpg.de) was employed to annotate the mitogenome using the reference mitogenome of *Lonicera japonica* (Accession No. MZ504724). All tRNA genes were identified with tRNAscan-SE using default settings (Lowe and Eddy, 1997). And a circular mitochondrial map was created using Organellar Genome DRAW (Greiner et al., 2019). The assembled and annotated mitogenome of *L. macranthoides* ‘Yulei 1’ was deposited in GenBank (Accession No. PQ472412).

### Analysis of repeats and recombination of mitogenome

REPuter was utilized to identify repeats using default parameters (Kurtz et al., 2001). The recombinant conformations rearranged by repeats were illustrated as described previously (Shearman et al., 2014). Simple sequence repeats (SSRs) in the genome were analyzed using the MISA program as previously reported (Thiel et al., 2003).

### Genome alignments

The *L. macranthoides* mitogenome was aligned with the *L. macranthoides* chloroplast genome, *L. macranthoides* nuclear genome, and the *L. japonica* mitogenome via BLASTN 2.9.0+ (Duan et al., 2024). Collinearity analysis was performed based on the following criteria: matching rate ≥ 95%, E-value ≤ 1e−6, and length ≥ 200. The alignment between the mitogenome of *L. macranthoides* and *L. japonica* was conducted using default parameters. Diagrams were created using TB-tools (Chen et al., 2020). The National Center for Biotechnology Information (NCBI) BioProject number for the *L. macranthoides* nuclear genome is PRJNA800599. The accession number for the *L. japonica* mitogenome is PP857827. The chloroplast genome of *L. macranthoides* (Yulei 1) was sequenced, assembled, and annotated for the first time in this study, following the methods outlined in a previous report (Miao et al., 2024). This chloroplast genome is also deposited in GenBank with Accession No. PQ472411.

### RNA editing sites prediction

To identify RNA editing sites, we followed the methods described previously (Lo Giudice et al., 2020). Three replicates of RNA sequencing were performed on the flower (the flower of ‘Yulei’ retains bud stage permanently, without flowering capability), stem, and leaf tissues from the *L. macranthoides* cultivar ‘Yulei 1’ (Accession No. SRR30317731). The protein-coding genes in the mitogenome were mapped to the transcriptomic data to predict RNA editing sites in different tissues.

### Phylogenetic analyses

Using the mitogenomic data available in public databases and principles of plant classification, we downloaded mitogenomes for phylogenetic tree construction. Twenty conserved mitochondrial PCGs (*atp4*, *atp6*, *atp8*, *atp9*, *ccmC*, *cox1*, *cox2*, *cox3*, *nad1*, *nad2*, *nad3*, *nad4*, *nad4L*, *nad5*, *nad6*, *nad7*, *nad9*, *rps3*, *rps12*, *rps13*) across these species were aligned in Muscle with default parameters (Edgar, 2004). After eliminating gaps and missing data, a maximum likelihood tree was constructed using IQ-TREE 2 with the GTR+F+R4 nucleotide substitution model (Lanfear et al., 2020). A bootstrap consensus tree was generated from 1,000 bootstrap replicates. Set *Ginkgo biloba*, *Zea mays*, and *Oryza sativa* as outgroups.

### ORFs prediction and amplification

All ORFs were identified using ORFfinder with a length threshold of > 300 bp (https://www.ncbi.nlm.nih.gov/orffinder/). A total of 349 ORFs were obtained based on the mitogenome of ‘Yulei 1’. Subsequently, these ORFs were further aligned with the mitogenome of L. japonica. Ninety-one unique ORFs in ‘Yulei 1’ were selected by removing shared ORFs. These ORFs were searched in the CCD database (http://www.ncbi.nlm.nih.gov/Structure/cdd/cdd.shtml), InterPro database (http://www.ebi.ac.uk/interpro/), and SMART database (http://smart.embl-heidelberg.de/) to predict their protein families, setting an identity threshold of > 95%. The coverage of the alignments of the seven selected ORFs was checked in the NCBI non-redundant protein database (https://blast.ncbi.nlm.nih.gov/). RNA sequencing data from flower, stem, and leaf tissues were used to determine transcribable ORFs. The transmembrane domains of ORFs were predicted using the TMHMM server version 2.0 (Krogh et al., 2001). The secondary and tertiary protein structures were predicted by AlphaFold2 (https://colab.research.google.com/github/sokrypton/ColabFold/blob/main/AlphaFold2.ipynb), the protein structures were visualized using EzMol (https://www.sbg.bio.ic.ac.uk/ezmol/). Primers used for the amplification of *orf125* were ATGCGCGTAGCTATTGCCTT and TTCATCCGGGAAAAGCCATCT. The 1.1×T3 Super PCR Mix (Tsingke, China) was used for amplification according to the manufacturer’s protocol.

## Results

### Features of the mitogenome of *L. macranthoides* ‘Yulei 1’

**Figure 1:**
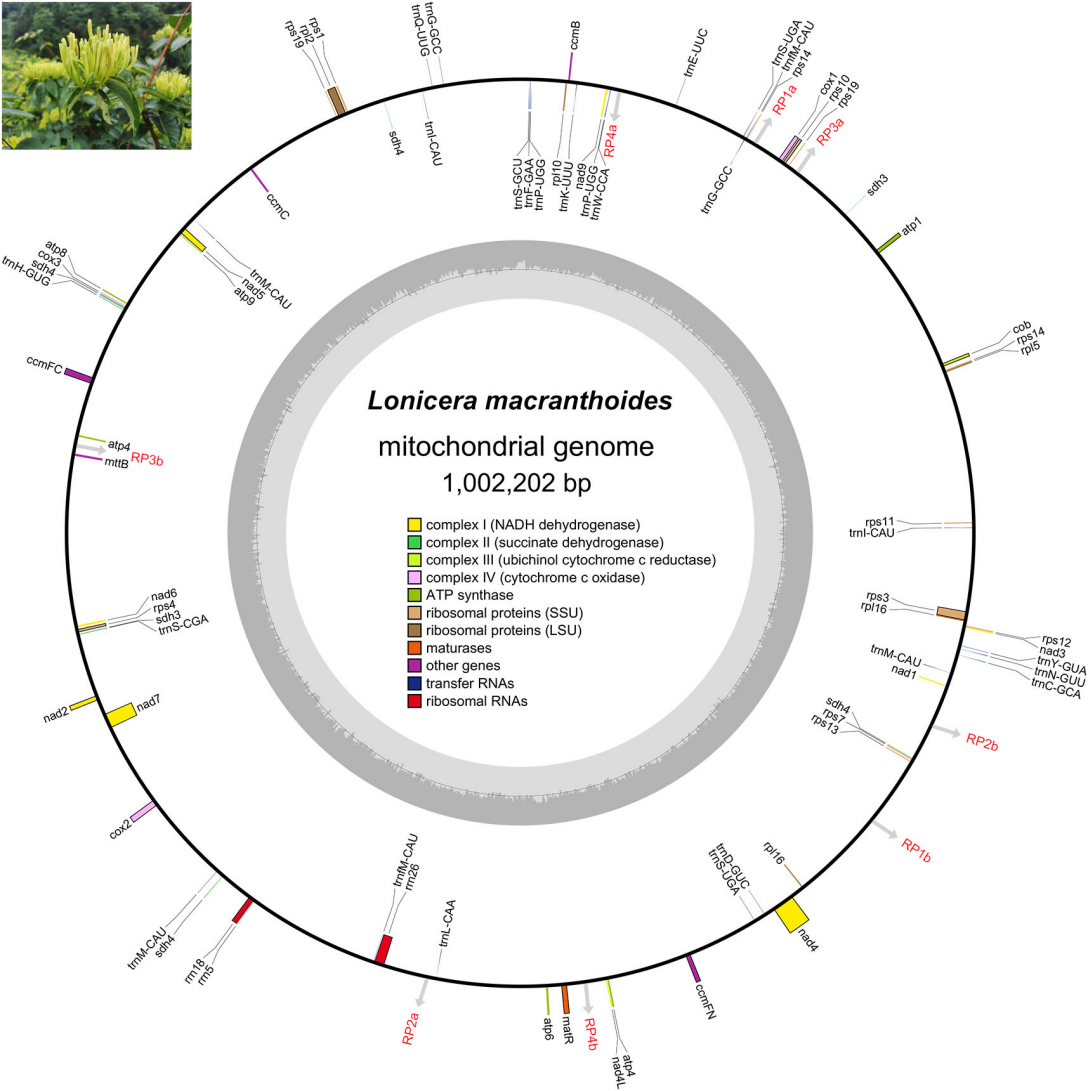
The annotated mitogenome scheme of *L. macranthoides* ‘Yulei 1’. The GE-Seq tool was employed to annotate the mitogenome. Genes listed inside and outside of the outer loop are transcribed clockwise and counterclockwise, respectively. The inner shaded loop depicted the GC content of the mitogenome in each section. The gray arrows indicate the positions of the dispersed repeats that mediate the rearrangements of the mitogenome. The image in the upper left corner shows bud-type flower of *L. macranthoides* cultivar ‘Yulei 1’, whose mitogenome was studied in this research.

The assembled mitogenome of *L. macranthoides* ‘Yulei 1’ is circular and has a length of 1,002,202 bp (**Figure 1**; **Supplementary Figure S1**). The nucleotide composition is 27.56% A, 27.59% T, 22.38% G, and 22.47% C, resulting in a total GC content of 44.85% (**Figure 1**; **Supplementary Table S2**). The mitogenome contains 48 PCGs, including 8 PCG copies, 3 rRNAs, and 19 unique tRNAs along with 7 tRNA copies. PCGs, rRNAs, and tRNAs represent 3.51%, 0.57%, and 0.19% of the entire mitogenome, respectively (**Table 1**; **Supplementary Table S2**). Among the 40 unique PCGs, 10 contain introns, with 8 exhibiting cis-splicing and 2 exhibiting trans-splicing (**Table 1**; **Supplementary Figures S2**-**S3**). No functional gene losses were observed in the mitogenome, including genes related to the ATP synthase complex and NADH dehydrogenase complex, which are critical for mitochondrial function.

**Table 1:** The coding genes in the mitogenome of *L. macranthoides* ‘Yulei 1’.

Group of genes	Name of genes
**Subunit of ATPase**	*atp1, atp4, atp4_copy2, atp6, atp8, atp9*
**Cytochrome C biogenesis**	*ccmB, ccmC, ccmFC* ^b^ *, ccmFN*
**Apocytochrome b**	*cob*
**Subunit of cytochrome C oxidase**	*cox1, cox2, cox3*
**Maturase R**	*matR*
**Transport membrane protein**	*mttB*
**Subunit of NADH dehydrogenase**	*nad1* ^a^ *, nad2* ^b^ *, nad3, nad4* ^b^ *, nad4L, nad5* ^a^ *, nad6, nad7* ^b^ *, nad9*
**Small subunit of ribosome**	*rps1, rps10* ^b^ *, rps11, rps12, rps13, rps14, rps14_copy2, rps19, rps19_copy2, rps3* ^b^ *, rps4, rps7*
**Large subunit of ribosome**	*rpl10, rpl16, rpl16_copy2, rpl2* ^b^ *, rpl5*
**Subunit of succinate dehydrogenase**	*sdh3, sdh3_copy2, sdh4, sdh4_copy2, sdh4_copy3, sdh4_copy4*
**Transfer RNAs**	*trnS-CGA, trnC-GCA, trnD-GUC, trnE-UUC, trnF-GAA, trnG-GCC, trnG-GCC_copy2, trnH-GUG, trnK-UUU, trnL-CAA, trnfM-CAU, trnM-CAU, trnM-CAU_copy2, trnM-CAU_copy3, trnfM-CAU_copy2, trnI-CAU, trnI-CAU_copy2, trnN-GUU, trnP-UGG, trnP-UGG_copy2, trnQ-UUG, trnS-GCU, trnS-UGA, trnS-UGA_copy2, trnW-CCA, trnY-GUA*
**Ribosomal RNAs**	*rrn18, rrn26, rrn5*

a, trans-splicing genes. b, cis-splicing genes.

The bold text represents the type of genes.

### Repeats and Recombination in the Mitogenome of *L. macranthoides* ‘Yulei 1’

**Figure 2:**
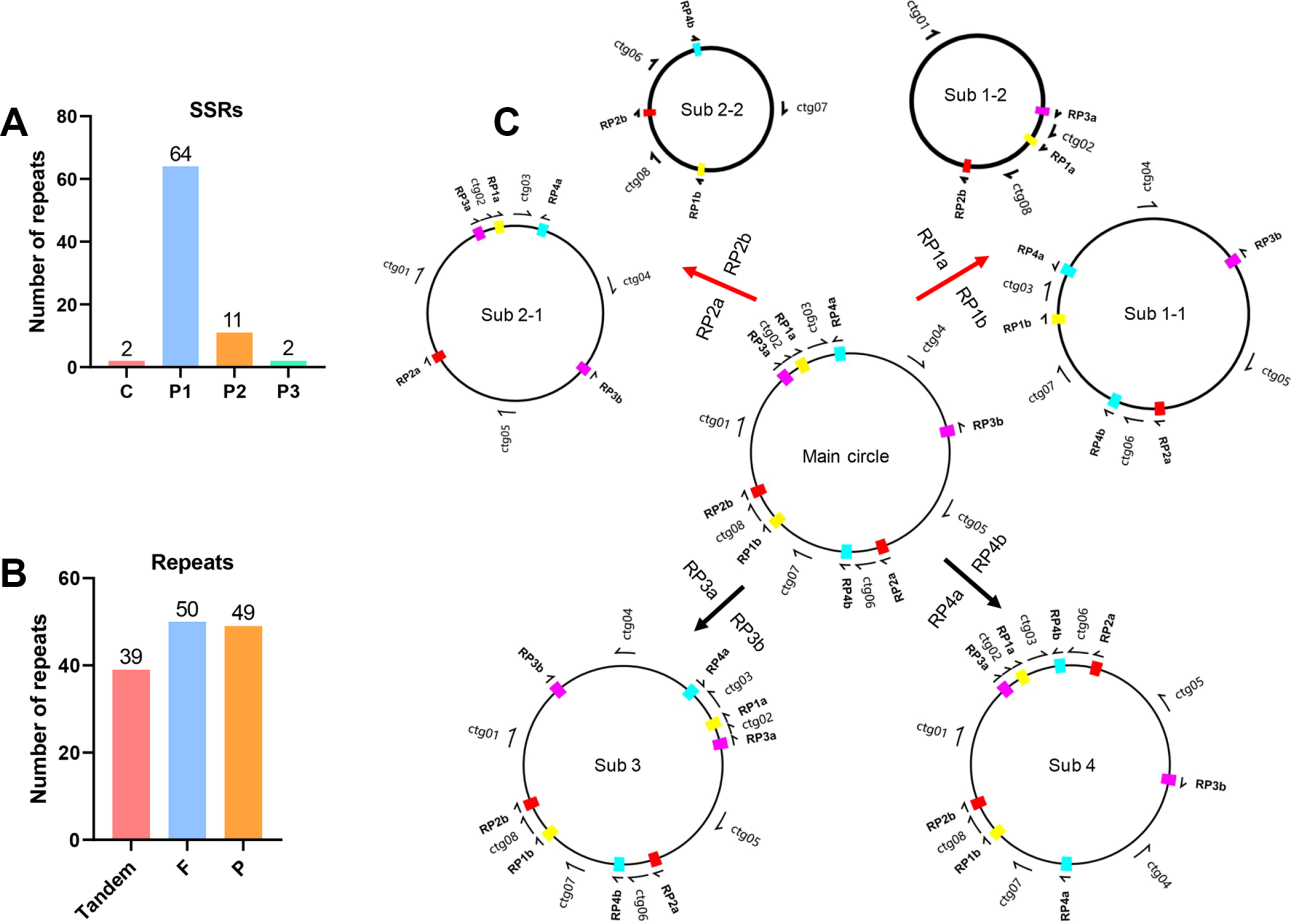
Repeats in the mitogenome of *L. macranthoides* ‘Yulei 1’. **(A)** The statistics of SSRs mitogenome of *L. macranthoides* ‘Yulei 1’. c represents compound SSRs. p1, p2 and p3 represent mono-, di-, and tri- nucleotide SSRs, respectively. **(B)** The count of tandem repeats and dispersed repeats. F, Direct repeats. P, Inverse repeats. **(C)** Mitogenome recombination mediated by RP1a/b, RP2a/b, RP3a/b and RP4a/b, respectively.

Seventy-nine SSRs were identified using the MISA program, which included 2 compound SSRs, 64 mononucleotide SSRs, 11 dinucleotide SSRs, and 2 trinucleotide SSRs (**Figure 2A**; **Supplementary Table S3**). Additionally, 39 tandem repeats and 99 dispersed repeats were detected using the REPuter tool, and 50 direct repeats and 49 inverse repeats were included in the dispersed repeats (**Figure 2B**; **Supplementary Tables S4**-**S5**). Further analysis indicated that four pairs of dispersed repeats contribute to the mitogenome recombination in *L. macranthoides* ‘Yulei 1’. Two pairs of direct repeats (RP1a/1b, RP2a/2b) lead to distinct sub-configurations derived from the main configuration, while two pairs of inverse repeats (RP3a/3b, RP4a/4b) facilitate contig inversions (**Figure 2C**; **Supplementary Table S6**). These recombination events significantly altered the structure of the mitochondrial chromosome. However, no coding genes were located at the recombination sites, suggesting minimal impact on the structure of functional genes (**Figures 1**, **2C**; **Supplementary Table S6**).

### Prediction of RNA editing sites

**Figure 3:**
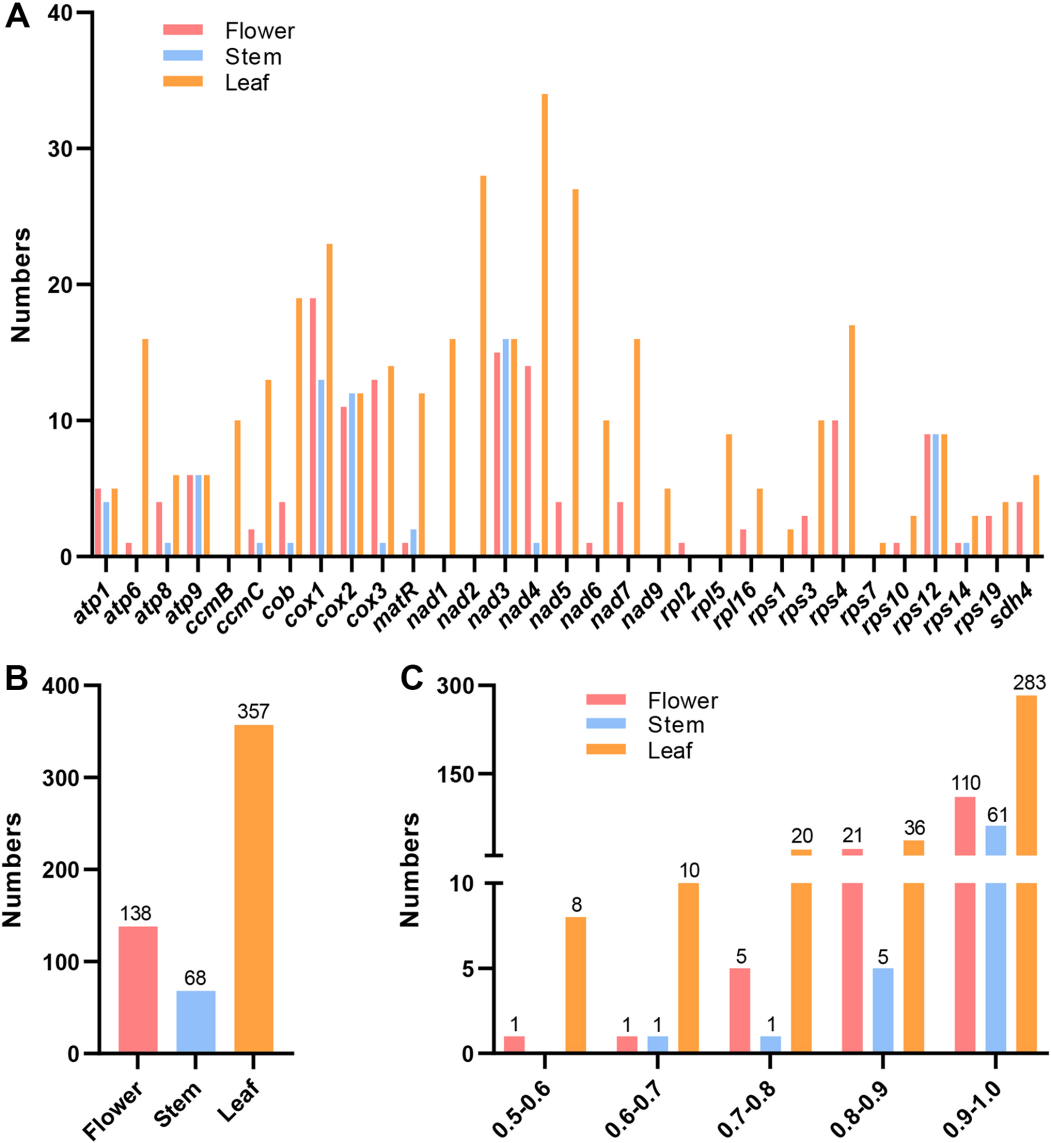
RNA editing events in the mitogenome of *L. macranthoides* ‘Yulei 1’. **(A)** RNA editing sites in each mitochondrial PCG of flower, stem and leaf, frequency > 0.5. **(B)** The total RNA editing sites of mitochondrial PCGs in flower, stem and leaf, frequency > 0.5. **(C)** The RNA editing frequency of mitochondrial PCGs of flower, stem and leaf, only editing events with frequency > 0.5 were retained.

RNA editing events are common in animals and plants to ensure accurate gene expression. And the RNA editing patterns were different in various tissues (Huntley et al., 2016). This study separately identified RNA editing sites of PCGs in the flower, stem, and leaf tissues of ‘Yulei 1’. Based on the annotated mitogenome, three replicate RNA-sequencing datasets across the three tissues were generated for analysis to capture all changes in PCG transcripts (**Supplementary Tables S7**-**S9**). With a threshold of RNA editing frequency > 0.5, 24 PCGs in flower, 13 PCGs in stem, and 29 PCGs in leaf exhibited RNA editing (**Figure 3A**). Leaf had the highest total RNA editing sites (357), followed by flower (138) and stem (68) (**Figure 3B**). The distribution of RNA editing frequency showed that most events occurred at frequencies above 0.9 in all tissues (**Figure 3C**). When the frequency was set to 1, ATP synthase genes (*atp1*, *atp8*, *atp9*), the cytochrome C biogenesis gene (*ccmC*), ubiquinol cytochrome C reductase (*cob*), and cytochrome C oxidase genes (*cox1*, *cox2*) exhibited RNA editing sites across all three tissues. Although the number and locations of editing sites varied among tissues (**Supplementary Tables S7**-**S9**). The small subunit ribosomal protein gene (*rps12*) had RNA editing sites only in the stem. While editing sites in the cytochrome C biogenesis gene (*ccmB*), NADH dehydrogenase genes (*nad1*, *nad2*, *nad9*), large subunit ribosomal protein gene (*rpl5*), and several small subunit ribosomal protein genes (*rps1*, *rps3*, *rps7*, *rps10*, *rps14*) were exclusive to leaf. No unique RNA editing events were identified in flower (**Figure 3A**; **Supplementary Tables S7**-**S9**). All identified editing events were C to U transitions, consistent with previously reported mitochondrial RNA editing biases (Miao et al., 2024).

### Identification of MTPTs and MTNUs

**Figure 4:**
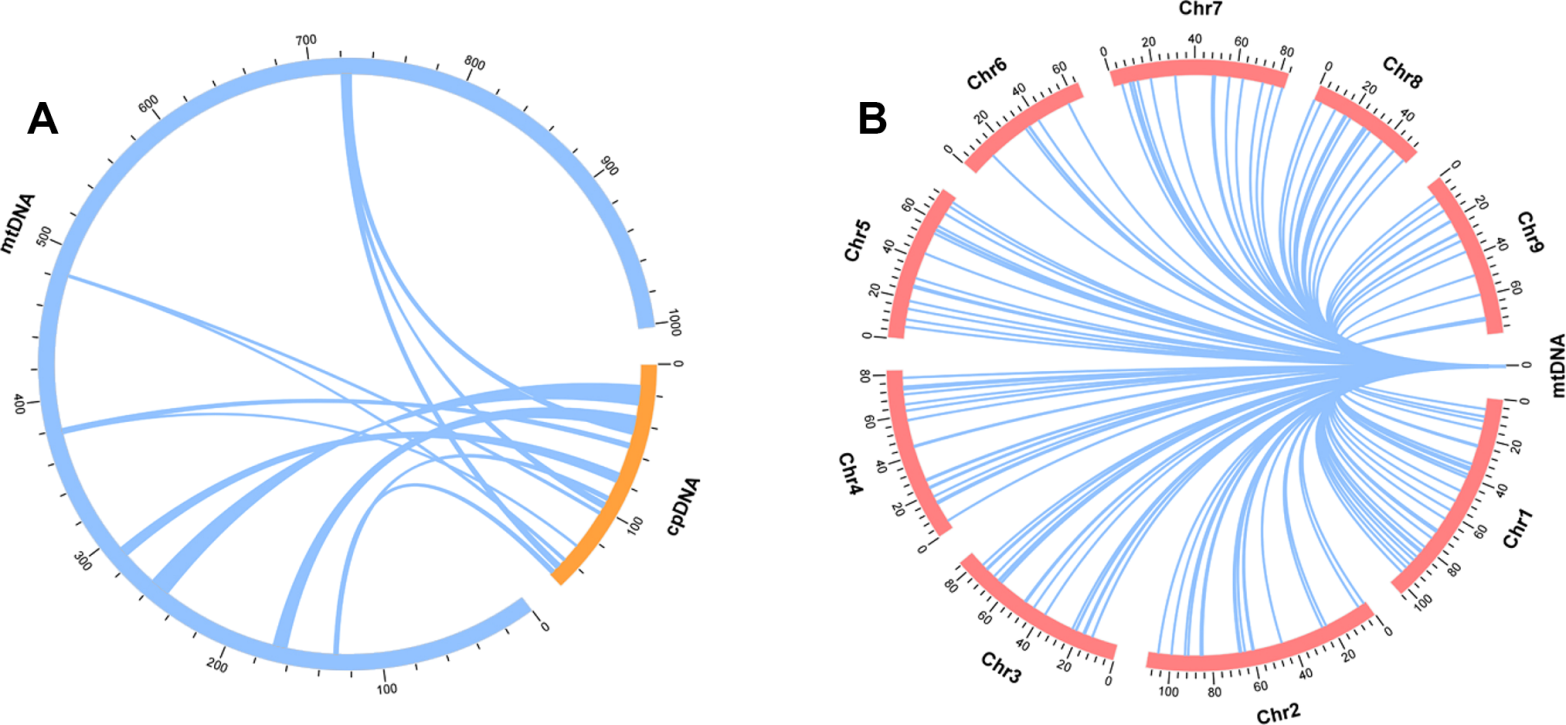
Detection of MTPTs and MTNUs in the mitogenome of *L. macranthoides* ‘Yulei 1’. **(A)** The sequences transferred from chloroplast, set sequence similar ≥ 95%. **(B)** The sequences shared between nuclear genome and mitogenome, sequence identity was set ≥ 95%.

To explore the transfer sequences between the mitogenome of *L. macranthoides* ‘Yulei 1’ and its chloroplast and nuclear genomes, a collinearity analysis was performed using BLASTN 2.9.0+. The chloroplast genome of *L. macranthoides* ‘Yulei 1’ was assembled, and 18 MTPTs (segments transferred from the chloroplast to the mitogenome) were determined. These MTPTs are 43,679 bp in total and account for 4.36% of the mitogenome (**Figure 4A**; **Supplementary Table S10**). Additionally, 226 MTNUs (fragments shared between the nuclear and mitogenome) were found via a reported nuclear genome (PRJNA800599). These fragments are totally 220,292 bp and account for 21.98% of the mitogenome (**Figure 4B**; **Supplementary Table S11**). Most of the transferred sequences were located in non-coding regions of the mitogenome. Shared coding sequences among the three genomes primarily included some tRNAs and *rps* genes. No other PCGs were found to be shared completely (**Supplementary Tables S10**-**S11**).

### Phylogenetic analysis of *L. macranthoides* based on mitogenome data

**Figure 5:**
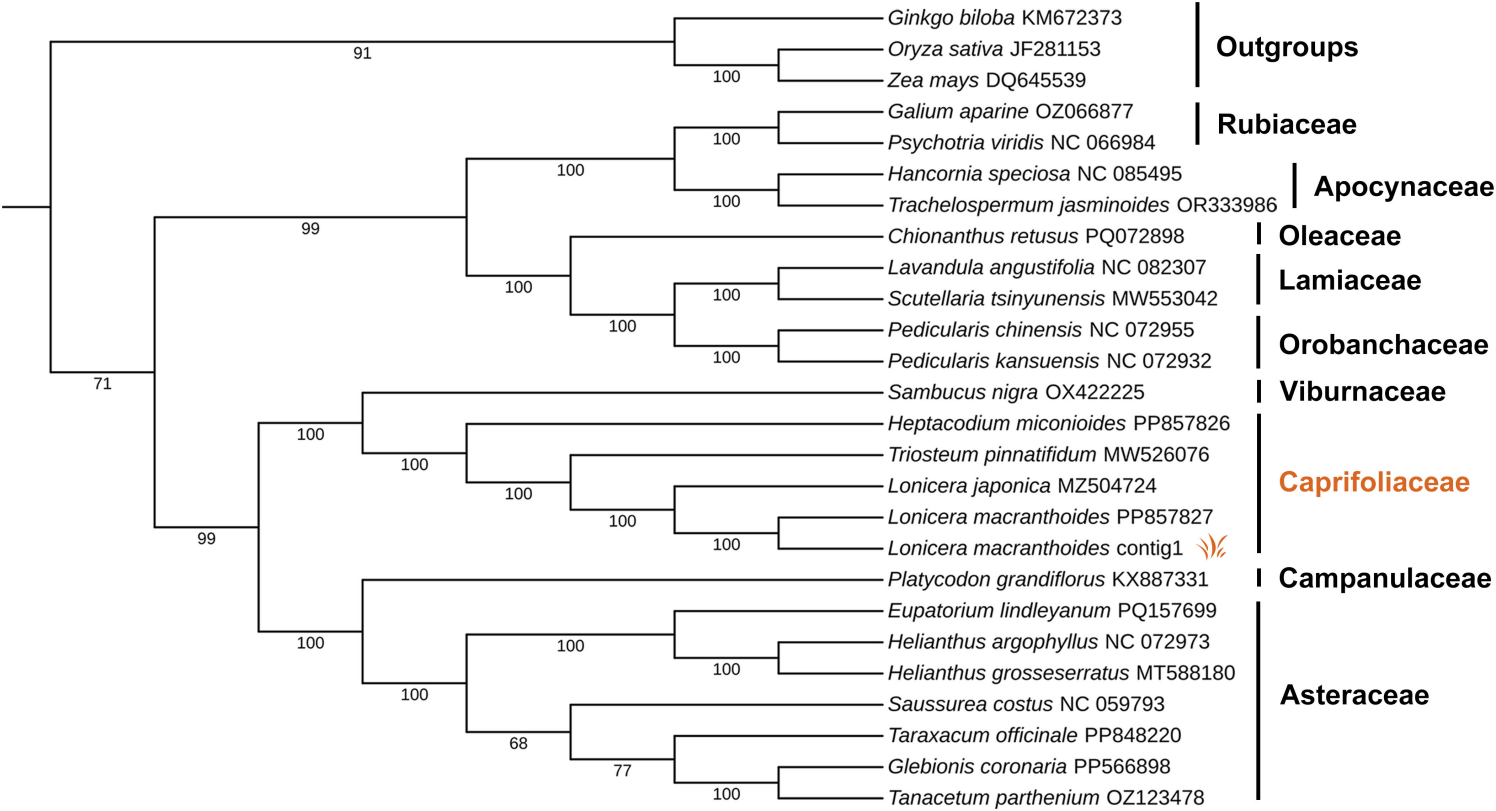
Phylogenetic tree constructed with mitogenomes of *L. macranthoides* and other 24 species. Maximum-likelihood phylogenetic tree based on 20 single copy orthologous genes shared among 25 species (**Supplementary Table S12**), *L. macranthoides* mitogenome with Accession No. PP857827 was downloaded as raw data from GenBank. *L. macranthoides* contig1 was assembled and annotated in this article. Set *Ginkgo biloba*, *Zea mays*, *Oryza sativa* as outgroups, the numbers on each node are bootstrap support values.

Twenty-five mitogenomes were retrieved from GeneBank (**Supplementary Table S12**), and 20 common PCGs were used to construct a maximum likelihood phylogenetic tree. The resulting tree demonstrated strong support, with 21 of 24 nodes having bootstrap values over 90%, including 18 nodes with 100% support (**Figure 5**). The analysis indicated that *L. macranthoides* is most closely related to *L. japonica*, and more distantly related to *Triosteum pinnatifidum* and *Heptacodium miconioides* within the Caprifoliaceae family. The phylogenetic tree also suggested a close relationship between Caprifoliaceae and Viburnaceae. It is consistent with their classification within Dipsacales. Overall, these results serve as a valuable foundation for subsequent phylogenetic affinity analyses of *Lonicera* species.

### ORFs found in the mitogenome of *L. macranthoides* ‘Yulei 1’

*L. macranthoides* cultivar ‘Yulei 1’ is male sterile. Its flowers remain in the bud stage with aborted pollen and lack flowering ability. To determine if this sterility is associated with aberrant mitochondrial function, we sought to compare its mitogenome with that of a fertile *L. macranthoides* cultivar. However, we only retrieved a complete mitogenome for *L. macranthoides* in GenBank (Accession No. PP857827, raw data and unpublished in any papers). Due to the loss of the description of the cultivar name, we could not ascertain the reproductive characteristics of the material used for the mitogenome assembly (PP857827). It is pointless to use this mitogenome at the level of sterility research. Therefore, we focused on the closely related species *L. japonica*, which is of flowering type and fertile. Our analysis revealed that approximately 84% of the nucleotides in the mitogenome of ‘Yulei 1’ match those of *L. japonica* (**Supplementary Figure S4**; **Supplementary Table S13**). In other words, the mitogenome of ‘Yulei 1’ only contains 16% unique nucleotide sequences when compared to *L. japonica*.

**Figure 6:**
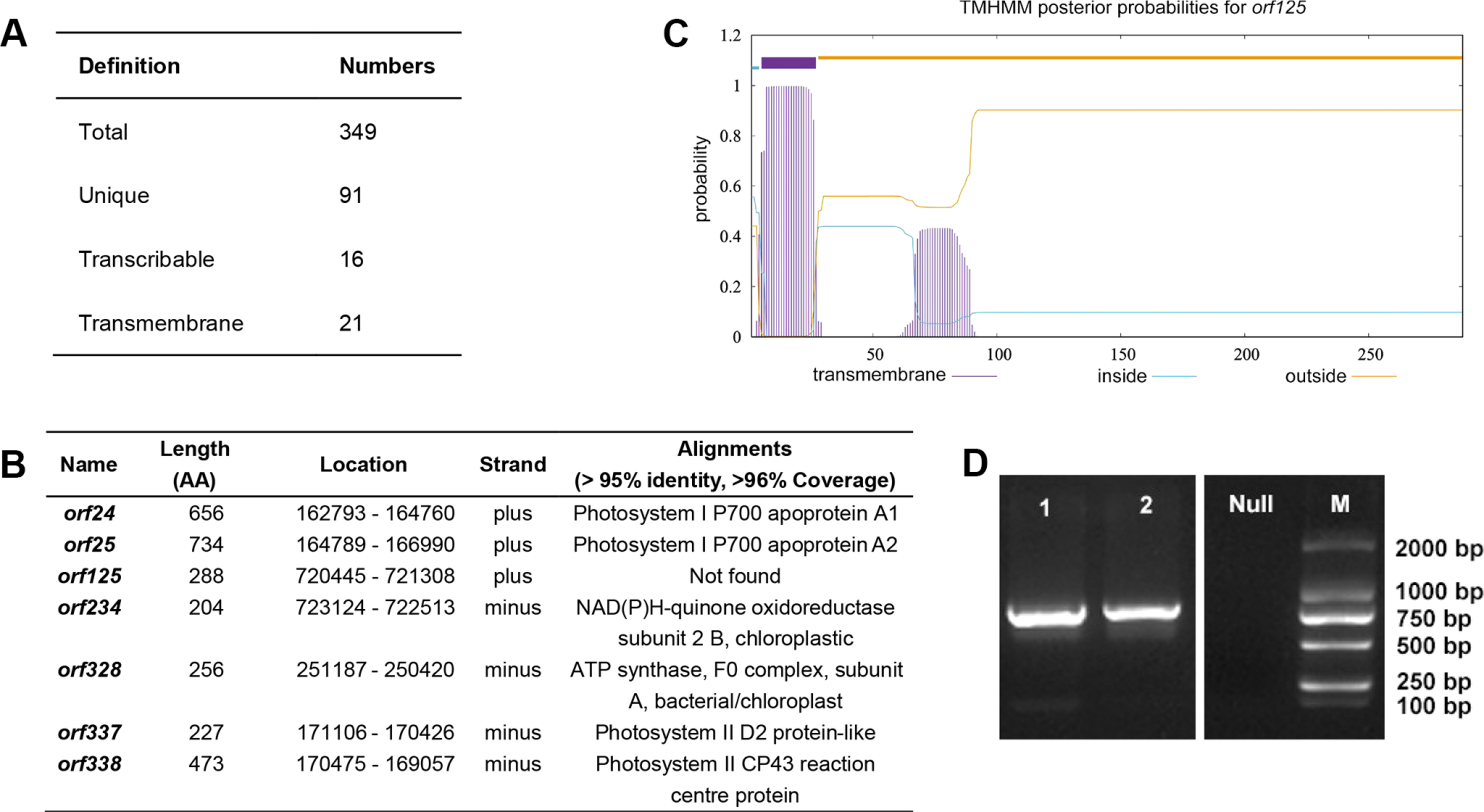
ORFs in the mitogenome of *L. macranthoides* ‘Yulei 1’. **(A)** Statistics of ORFs in the mitogenome of *L. macranthoides* ‘Yulei 1’. **(B)** The 7 ORFs with transmembrane domain and transcriptional ability in mitogenome of *L. macranthoides* ‘Yulei 1’. AA, amino acids. The amino acid sequences of all ORFs were blast in CCD database, InterPro database and SMART database with > 95% identity. The coverage of ORFs in the alignments was checked in the NCBI non-redundant protein database. The coverage percentage of all ORFs was over 96%, except for orf125. **(C)** The putative transmembrane domain of orf125. **(D)** The amplification of *orf125*, line 1 represents amplified products in ‘Yulei 1’, line 2 represents amplified products in wild type of *L. macranthoides*, Null is negative control, M represents marker BL2000.

ORFs are potential transcripts with specific molecular functions in the mitogenome. In most cases, some abnormal ORFs are toxic to mitochondria and related to CMS (Horn et al., 2014). To characterize these sequences, we identified ORFs longer than 300 bp in the ‘Yulei 1’ mitogenome using ORFfinder. Totally, 349 ORFs were determined (**Figure 6A**; **Supplementary Table S14**). After removing co-shared ORFs in the mitogenome of *L. japonica* through BLAST analysis, we identified 91 unique ORFs in the mitogenome of ‘Yulei 1’ (**Supplementary Table S14**). Combining transcriptome data in the flower and bioinformatics analysis, we found 16 ORFs were transcribable and 21 ORFs possessed transmembrane domains. In particular, 7 ORFs were inferred to have transcriptional ability along with transmembrane domains (**Figure 6B**).

All ORFs were queried against the CCD, InterPro, and SMART databases with > 95% identity. Most ORFs did not match any known protein domains. Fifteen of 16 transcribable ORFs matched photosystem proteins and DNA-directed RNA polymerase subunit beta. It is indicated that most of them may be chloroplast- and nucleus-derived proteins. The ORFs with transmembrane domains were usually associated with CMS (Cheng et al., 2021). Notably, among the 7 transcribable ORFs with transmembrane domains, 6 encoded chloroplast-related proteins (**Figures 6B, C**), which are known to enhance electron transfer (Caffarri et al., 2014; Kühlbrandt, 2019). Only *orf125* had no hits of known protein domains in any of the databases. Further analysis of its protein structure indicated that orf125 is different from the protein structures of the mitochondrial membrane proteins, such as atp6 and cox3. The orf125 protein exhibits stronger hydrophilicity on its surface, while the mitochondrial membrane proteins atp6 and cox3 possess stronger hydrophobicity (**Supplementary Figure S5**). Structurally, this indicates that orf125 does not have functional competition with the mitochondrial membrane proteins. Most importantly, *orf125* was detected in both ‘Yulei 1’ and the fertile wild type of *L. macranthoides* (**Figure 6D**). This suggests that *orf125* is unlikely to be responsible for the sterility of ‘Yulei 1’. On the basis of the above findings, it is indicated that the unique sequences in the mitogenome of ‘Yulei 1’ compared to *L. japonica* do not account for its sterility.

## Discussion

Genome sequencing facilitates comprehensive studies of living organisms. Mitochondria are essential for energy production in plants, they are semi-autonomous and inherited maternally (Gray et al., 1999; Li et al., 2021). Nearly 2,000 plant mitogenomes have been deposited in GenBank (https://www.ncbi.nlm.nih.gov/). However, reports on the complete mitogenomes of the Caprifoliaceae family remain limited. Especially, the mitogenome of *L. macranthoides* has not been analyzed yet. In this study, we obtained the complete mitogenome of *L. macranthoides* ‘Yulei 1’. Similar to previously reported mitogenomes (Cheng et al., 2021; Li et al., 2022), the coding sequences comprise only 4.27% of the mitogenome, the majority of the mitogenome consists of non-coding regions. These non-coding regions likely arise from sequence duplication, sequence transfer from chloroplast, and gene exchanges with the nuclear genome, which are important evolutionary drivers for mitogenomes (Cho et al., 2004; Walker and Moraes, 2022). Repeats are important for developing genetic markers in population and evolutionary analyses. In this study, we identified 79 SSRs, 39 tandem repeats, and 99 dispersed repeats. It provides a basis for developing genetic markers to differentiate various *L. macranthoides* cultivars and conduct population or evolutionary studies. Dispersed repeats are typically involved in mediating mitogenome recombination (Maréchal and Brisson, 2010). For example, the direct repeats DRa/b caused the main circular mitogenome of *Scutellaria tsinyunensis* to split into two sub-circular conformations (Li et al., 2021). Fang et al. found that six inverse repeats and three direct repeats could lead to rearrangements of the mitogenome in *Prunus salicina* (Chinese Plum) (Fang B. et al., 2021). In our study, we identified two direct repeats (RP1a/b, RP2a/b) and two inverse repeats (RP3a/b, RP4a/b) that could induce mitogenome rearrangements. It is indicated that multiple conformations and active rearrangements exist in the mitogenome of *L. macranthoides* ‘Yulei 1’.

RNA editing events usually occur in the processes of tissue development, cell signaling transduction and stress tolerance (Tang and Luo, 2018). It is a crucial post-transcriptional modification that significantly impacts the function of genes. For example, RNA editing in the *atp6* gene can cause its premature termination of translation, which may lead to pollen abortion in cotton (Suzuki et al., 2013). At least five protein families involve in the regulation of RNA editing. These include PPRs (pentatricopeptide repeat) protein, MORFs (multiple organellar RNA editing factors)/RIPs (RNA editing factor interacting proteins), ORRM1 (organelle RNA recognition motif), OZ1 (organelle zinc-finger) and ADAR (adenosine deaminase acting on RNA) (Qulsum et al., 2019; Li et al., 2023). The PPRs are the common regulator of RNA editing events in plants, and they usually directly bind to their cognate RNA to modify RNA (Qulsum et al., 2019). The previous researches were mainly developed on the basis of nuclear genome and chloroplast genome, or the authors primarily focused on the genes in a special tissue. Numerous RNA editing events have also been reported in plant mitochondria (Handa, 2003; Fang B. et al., 2021). Fang et al. found that RNA editing events of mitogenome are different in the leaves and roots of tobacco (*Nicotiana tabacum*), their research preliminarily revealed the phenomenon that RNA editing events in the mitochondrial genome are variable among plant tissues (Fang J. et al., 2021). However, specific RNA editing events across various tissues of plant mitogenomes are still rarely discussed (Miyata and Sugita, 2004; Qulsum et al., 2019), likely due to the relatively limited mitogenomic data available.

In this study, we first sequenced the mitogenome of *L. macranthoides* ‘Yulei 1’ and performed RNA-sequencing analyses on flower, stem, and leaf tissues. Using the RNA-editing site finder REDItools, the co-shared RNA editing sites across three replicates of RNA-sequencing data were identified. We found that RNA editing events differed among the three tissues. Leaves had the highest total of 357 RNA editing sites, followed by flowers with 138, and stems with 68. In total, 29 PCG mRNAs were edited in leaves, 24 in flowers, and only 13 in stems. Notably, specific genes exhibited distinct editing patterns. No specific genes were edited in flowers compared to leaves and stems. However, *rps12* was edited only in stems. While *ccmB*, *nad1*, *nad2*, *nad9*, *rpl5*, *rps1*, *rps3*, *rps7*, *rps10*, and *rps14* were edited only in leaves. RNA editing of coding genes in different tissues plays vital roles in tissue development and function (Tang and Luo, 2018). For instance, leaves are the primary sites for energy supply and carbon fixation, where frequent gene expression and intense sunlight can damage genetic material (Caffarri et al., 2014; Xiong et al., 2017). RNA editing may repair these damages, ensuring genes function correctly (Small et al., 2019). Thus, it is not surprising that mitochondrial genes in leaves have the highest editing rates. This highlights the need to consider tissue specificity when studying mitochondrial RNA editing of coding genes.

The phylogenetic relationship of *L. macranthoides* has been analyzed at the nuclear genome level and the chloroplast genome level. The nuclear genome evolution analysis indicated that *L. macranthoides* and *L. japonica* consistently went through a common gamma whole-genome triplication (γ-WGT) event and a special whole-genome duplication (WGD) event. They were diverged till 1.30–2.27 million years ago (MYA). In dicotyledonous plants, the *Lonicera* genus (*L. macranthoides* and *L. japonica*) was the closest relative to the ancestor of *Daucus carota* (Yin et al., 2023). The phylogenetic analysis by chloroplast genome demonstrated that *L. macranthoides* clustered together with *L. japonica*. And *Lonicera* showed a closer relationship with *Triostrum* on the basis of reported data (Hu et al., 2018). However, the plant species in the reported phylogenetic trees were relatively too few to clarify the phylogenetic relationships comprehensively. And it has lacked a phylogenetic analysis at the level of mitogenome till now. Based on the first featured mitogenome of *L. macranthoides*, we constructed a phylogenetic tree containing 25 plant species. These outnumber the plant species used in phylogenetic tree construction of nuclear genomes (13 plant species) and chloroplast genomes (12 plant species). The constructed tree confirmed that *L. macranthoides* is most closely related to *L. japonica*. And *Lonicera* was closer to *Triostrum* than *Heptacodium* within the Caprifoliaceae family. This further clarified the phylogenetic relationships of *Lonicera* genus and Caprifoliaceae species.

‘Yulei 1’ is a male sterile cultivar of *L. macranthoides*. According to Xu, the pollen morphology of ‘Yulei 1’ is normal compared to fertile varieties. MTT (thiazolyl blue) staining assays indicated that the pollen of ‘Yulei 1’ is viable. However, it fails to germinate *in vitro*, which is the main reason for its sterility. Hybridization assays demonstrated that the pistil of ‘Yulei 1’ is fully fertile. Based on these results, Xu suggested that ‘Yulei 1’ may be male sterile and self-incompatible (Xu, 2016). CMS, genic male sterility (GMS), and nucleo-cytoplasmic male sterility are types of male sterility (Chen and Liu, 2014). CMS is often caused by functional defects in chloroplasts or mitochondria (Chase, 2007). Given the mitogenome of ‘Yulei 1’, we sought to explore whether its sterility is caused by mitochondrial defects.

Three factors can contribute to CMS at the mitochondrial level: (1) functional gene loss, (2) aberrant RNA editing, and (3) accumulation of toxic protein products (Chen et al., 2017). Our analysis of the ‘Yulei 1’ mitogenome found no loss of functional genes. Furthermore, no coding genes were located in the recombination regions. This indicates that mitogenome arrangements have minimal impact on coding genes. RNA editing in ATP synthase genes is typically associated with CMS. In CMS lines of *Sorghum bicolor* and rice (WA), no RNA editing or inefficient RNA editing was observed in *atp6* and *atp9* (Horn et al., 2014; Tang and Luo, 2018). In CMS lines of cotton (line D8CMS8518) and Yunnan purple rice (line Ying xiang A), the termination codon of the ATP synthase gene is aberrant because of the RNA editing events (Wei et al., 2010; Suzuki et al., 2013). These are the two main reasons that RNA editing events lead to CMS. In our study, RNA editing sites were detected in all *atp* genes in the flowers of ‘Yulei 1’. The resulting amino acid substitutions included Ser to Leu, Pro to Leu, Leu to Phe, Pro to Ser, and Ser to Phe. There were no premature terminations in the RNA editing events of *atp* genes in the flowers of ‘Yulei 1’. Therefore, RNA editing is unlikely to contribute to the CMS of ‘Yulei 1’.

ORFs are potential coding sequences in the mitogenome (Shearman et al., 2014). Many reports indicate that CMS-related ORFs can be toxic to the mitochondrial membrane (Chen and Liu, 2014; Chen et al., 2017). These ORFs often chimerically associate with *atp*, *cox*, and *nad* coding genes and feature transmembrane domains (Dewey et al., 1987; Cheng et al., 2021). In this study, we identified a total of 349 ORFs longer than 300 bp. Comparing to the mitogenome of *L. japonica*, we found 91 unique ORFs in ‘Yulei 1’, which were further analyzed using the InterPro, SMART, and CCD databases to identify functional domains. We identified seven transcribable ORFs with transmembrane domains in conjunction with RNA-sequencing data from flowers. Further analysis revealed that six of these ORFs (*orf24*, *orf25*, *orf234*, *orf328*, *orf337*, and *orf338*) were derived from chloroplasts. *orf125* could not be characterized in any of the databases, but we found it also present in the fertile wild type of *L. macranthoides*. It suggested that *orf125* is unlikely to contribute to the sterility of ‘Yulei 1’. In this study, we determined all detectable ORFs in the remaining mitogenomic sequences of ‘Yulei 1’, except that 84% of its mitogenomic sequences were in accordance with those of *L. japonica*. However, no classic CMS-associated ORFs were found, such as *atp*, *cox* chimeric ORFs or other special toxic ORFs. Overall, our findings suggest that the male sterility of ‘Yulei 1’ is not CMS caused by the mitochondria independently, and it may be due to GMS, nucleo-cytoplasmic male sterility, or self-incompatibility.

## Conclusions

In summary, we first obtained the mitogenome of ‘Yulei 1’, a male sterile cultivar of *L. macranthoides*. We comprehensively studied its features, including mitogenome structure, arrangements, RNA editing events, identification of MTPTs and MTNUs, phylogenetic analysis, and detection of ORFs. Importantly, we found that RNA editing events in the mitogenome varied among flower, stem, and leaf tissues, combining the mitogenome data with RNA sequencing results. Additionally, we focused on the possible relationship between CMS and the mitogenome of ‘Yulei 1’. Our analysis indicates that the sterility of ‘Yulei 1’ is unlikely to be caused by mitochondrion independently. This highlights the need to consider GMS and other factors. Overall, our data enrich the mitogenome resources for *L. macranthoides* within the Caprifoliaceae family. And they provide a foundation for future phylogenetic classification and molecular breeding efforts.

## Data Availability

The datasets presented in this study can be found in online repositories. The names of the repository/repositories and accession number(s) can be found in the article/**Supplementary Material**.
