# Supplementary material for: Mitochondrial genome of Lonicera macranthoides: features, RNA editing, and insights into male sterility
Source: Front Plant Sci. 2025 Jan 10;15:1520251. doi: 10.3389/fpls.2024.1520251 (PMC11759266; doi:10.3389/fpls.2024.1520251)
Supplement: Supplementary file 1 [file DataSheet1.docx]

**Supplemental figures**

**Figure S1** The raw assembly and BLASTn results graphs of mitochondrial sequencing data of *L. macranthoides* ‘Yulei 1’*.*

**Figure S2** Cis-splicing genes in the mitogenome of *L. macranthoides* ‘Yulei 1’*.*

**Figure S3** Trans-splicing genes in the mitogenome of *L. macranthoides* ‘Yulei 1’*.*

**Figure S4** Alignment of mitogenomes between *L. macranthoides* ‘Yulei 1’ and *L. japonica*.

**Figure S5** The protein structures of orf125 and two classic mitochondrial membrane proteins.


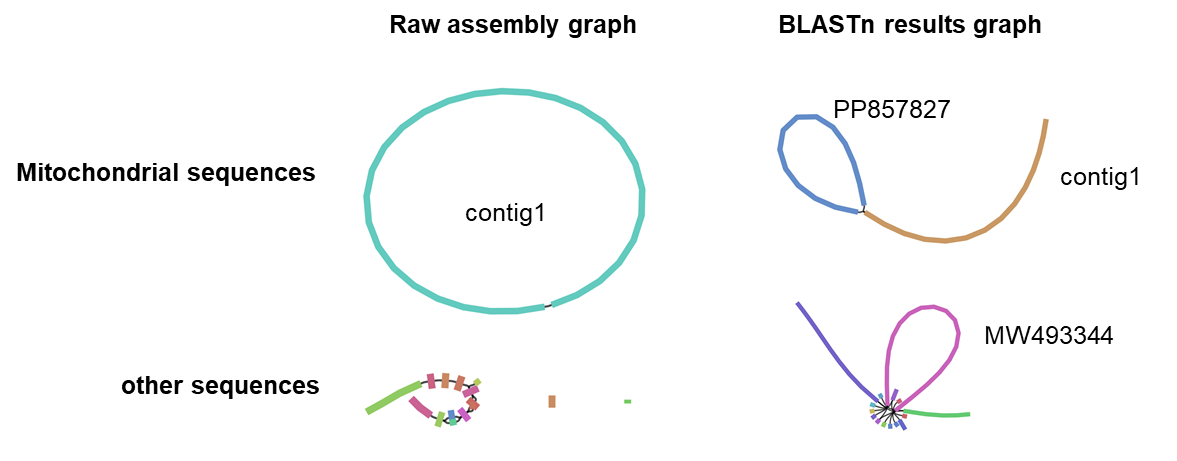


**Figure S1** The raw assembly and BLASTn results graphs of mitochondrial sequencing data of *L. macranthoides* ‘Yulei 1’.

The raw assembly and BLASTn results graphs were drawn by Bandage (Wick et al., 2015). As shown in the raw assembly graph, a circularized mitogenome without gaps (contig1) was successfully assembled. Only a few short sequences (other sequences) were not assembled onto the circular genome. After blast analysis, it was discovered that our assembly mitogenome (contig1) of ‘Yulei 1’ was closely blasted to the deposited mitogenome (Accession No. PP857827) of *L. macranthoides*. And the other sequences were closely blasted to the chloroplast genome (Accession No. MW493344) of *L. macranthoides*.

**Figure S2** Cis-splicing genes in the mitogenome of *L. macranthoides* ‘Yulei 1’*.*

**Figure S3** Trans-splicing genes in the mitogenome of *L. macranthoides* ‘Yulei 1’*.*


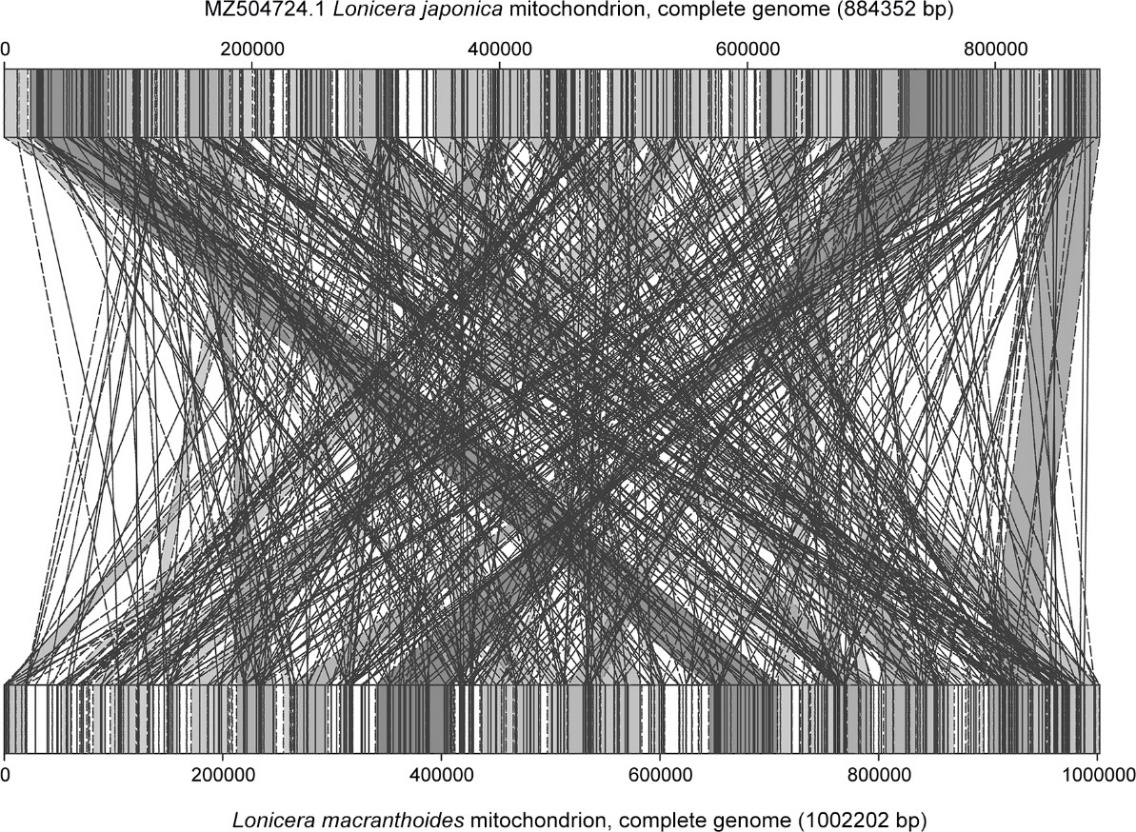


**Figure S4** Alignment of mitogenomes between *L. macranthoides* ‘Yulei 1’ and *L. japonica*.

There is no available mitogenome of the fertile cultivar of *L. macranthoides*. Therefore, we used the mitogenome of *L. japonica*, which is fertile and has the closest genetic relationship with *L. macranthoides*, to compare with the mitogenome of ‘Yulei 1’ to explore the causes of its male sterility. The alignment results indicated that there are 84% identical sequences between the mitogenomes of *L. japonica* and ‘Yulei 1’. Only 16% unique sequences exist in the mitogenome of ‘Yulei 1’. Our study detected all transcribable ORFs in the 16% unique sequences and evaluated the relationships between these ORFs and the male sterility. However, we could not detect the SNPs and non-coding RNAs in these 16% unique sequences to solve the puzzle of the male sterility of ‘Yulei 1’, because of the limitations of materials. The possibility of these factors resulting in mitochondrial functional abnormalities is rather low. Besides, SNPs and non-coding RNAs are usually associated with nucleo-cytoplasmic male sterility. Nevertheless, we cannot ignore their potential influence. In the present study, we are indeed unable to eliminate the possibility that these factors might contribute to the male sterility of *L. macranthoides*. This is a drawback of this research, and there is still a long way to go in studying this issue.


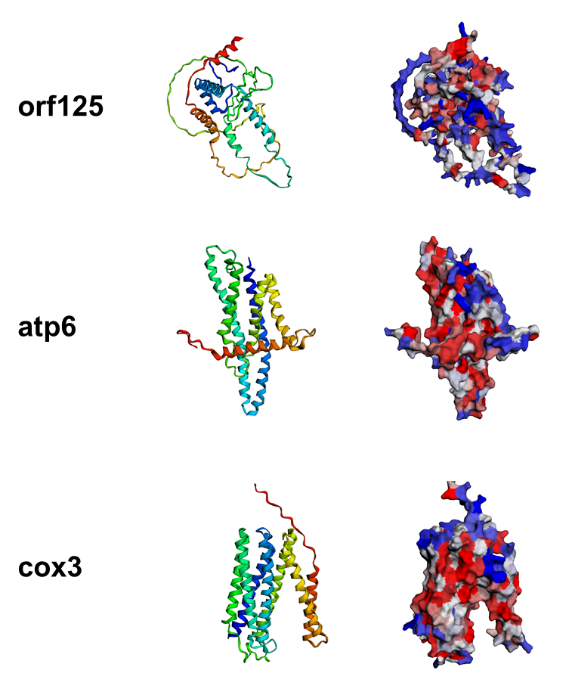


**Figure S5** The protein structures of orf125 and two classic mitochondrial membrane proteins.

In the protein structures shown in the left panel, the N-terminus to the C-terminus is marked from red to blue. In the protein structures shown in the right panel, the hydrophobic surfaces are marked in red, while the hydrophilic surfaces are marked in blue.
